# Ammonia Storage by Reversible Host–Guest Site Exchange in a Robust Metal–Organic Framework

**DOI:** 10.1002/anie.201808316

**Published:** 2018-10-01

**Authors:** Harry G. W. Godfrey, Ivan da Silva, Lydia Briggs, Joseph H. Carter, Christopher G. Morris, Mathew Savage, Timothy L. Easun, Pascal Manuel, Claire A. Murray, Chiu C. Tang, Mark D. Frogley, Gianfelice Cinque, Sihai Yang, Martin Schröder

**Affiliations:** ^1^ School of Chemistry University of Manchester Oxford Road Manchester M13 9PL UK; ^2^ ISIS Neutron and Muon Source Rutherford Appleton Laboratory Harwell Oxford Didcot OX11 0QX UK; ^3^ Diamond Light Source Harwell Science and Innovation Campus Oxfordshire OX11 0DE UK; ^4^ School of Chemistry Cardiff University Cardiff CF10 3XQ UK

**Keywords:** ammonia, metal–organic framework, MFM-300, neutron diffraction, storage materials

## Abstract

MFM‐300(Al) shows reversible uptake of NH_3_ (15.7 mmol g^−1^ at 273 K and 1.0 bar) over 50 cycles with an exceptional packing density of 0.62 g cm^−3^ at 293 K. In situ neutron powder diffraction and synchrotron FTIR micro‐spectroscopy on ND_3_@MFM‐300(Al) confirms reversible H/D site exchange between the adsorbent and adsorbate, representing a new type of adsorption interaction.

Approximately 150 million tonnes of NH_3_ (ammonia) was produced in 2017, making it one of the most important base chemicals in the world.^1^ As an energy resource, NH_3_ has an excellent hydrogen density with the hydrogen density of liquid NH_3_ and a 200‐bar H_2_ cylinder being 108 and 14 g L^−1^, respectively. It also has a high octane number and a low flame temperature, and its combustion to N_2_ and H_2_O is potentially environmentally benign.^2^ As a transportation fuel, NH_3_ can be incorporated into existing technologies such as internal combustion engines and gas turbines, but it also holds promise for renewable energy generation through H_2_ and NH_3_ fuel cells.^2^, ^3^ However, under ambient conditions, NH_3_ is a toxic and highly corrosive gas making it difficult to handle and store. For it to be transported in an energy efficient manner, NH_3_ is often liquefied to maximise its storage density through pipelines and in storage tanks.^4^ Liquid NH_3_ can be stored at ambient pressure at 240 K, whereas smaller quantities tend to be stored in pressurised vessels at 16–18 bar.4a Reducing or eliminating the energy consumption involved in NH_3_ storage is highly desirable, but any prospective material needs to show a high packing density that maximises NH_3_ storage amount within a given volume. It also needs to be capable of undergoing multiple cycles whilst retaining its adsorption capabilities. Zeolites, activated carbons, mesoporous silica and organic polymers have been tested for NH_3_ storage; however, these materials generally show low and/or irreversible uptakes.^5^

Constructed from metal ions and organic ligands, porous metal–organic frameworks (MOFs) are emerging solid sorbents for a wide variety of substrates.^6^ The highly porous nature of MOFs, coupled with their large surface areas (up to 7000 m^2^ g^−1^) and high concentration of binding sites, makes them promising candidates for gas storage. Indeed, extensive research efforts have been devoted to studying their capability to serve as H_2_, CH_4_ and CO_2_ stores.^7^ However, the potential of utilising MOFs for adsorption of corrosive and toxic gases remain poorly explored,^8^ primarily due to the limited stability of many MOFs. Recently, a number of stable MOFs have been tested for NH_3_ adsorption with a majority showing structural degradation on exposure or desorption.^9^ Here, we examine the adsorption, binding and reversible storage of NH_3_ in ultra‐stable MFM‐300(Al). At 273 K and 1.0 bar, MFM‐300(Al) shows an NH_3_ uptake of 15.7 mmol g^−1^ {corresponding to a formula of [Al_2_(OH)_2_(L)](NH_3_)_6.5_} leading to a packing density of 0.70 g cm^−3^, comparable to the liquid density of NH_3_ (0.681 g cm^−3^) at 240 K. At 293 K, MFM‐300(Al) also exhibits an impressive packing density of NH_3_ at 0.62 g cm^−3^, higher than leading MOFs and other state‐of‐the‐art porous materials, with an uptake of 13.9 mmol g^−1^. Importantly, the NH_3_ uptake in MFM‐300(Al) is fully reversible under conventional pressure‐swing conditions, and no loss of storage capacity was observed after 50 cycles of adsorption–desorption at 293 K. We have also employed in situ neutron diffraction, high resolution synchrotron X‐ray diffraction and micro‐FTIR spectroscopy for determination of the host–guest binding interaction at the molecular level. We have characterised a novel reversible host–guest site exchange mechanism that is intermediate between traditional physisorption and chemisorption.

MFM‐300(Al), [Al_2_(OH)_2_(L)], comprises of [AlO_4_(OH)_2_] moieties bridged by 3,3′,5,5′‐biphenyl‐tetracarboxylic acid (H_4_L) to afford a rigid “wine‐rack” framework with channels of ≈6.5 Å in diameter and hydroxyl groups pointing directly into the pore.^10^ MFM‐300(Al) has demonstrated exceptional adsorption and stability towards corrosive SO_2_ and NO_2_.^10^, ^11^ The adsorption isotherms for NH_3_ in MFM‐300(Al) were measured at 273–303 K, where a total uptake of 15.7 and 13.9 mmol g^−1^ was recorded at 273 and 293 K, respectively, at 1.0 bar (Figure 1). Due to the reactive nature of NH_3_, many materials that have been investigated previously are unstable to exposure and suffer significant degradation (Table S7). CoHCC {Co[Co(CN)_6_]_0.60_},9a [Co_2_Cl_2_(BBTA)] [BBTA=1*H*,5*H*‐benzo(1,2‐*d*),(4,5‐*d′*)bistriazole)],9c COF‐10,^12^ Amberlyst 15,5a 13X zeolite5a and MCM‐415a are the best‐performing porous materials from their respective categories that are stable to repeated NH_3_ exposure, with reported uptakes of 21.9 mmol g^−1^, 18.0 mmol g^−1^, 15.0 mmol g^−1^, 11.3 mmol g^−1^, 9.30 mmol g^−1^ and 7.90 mmol g^−1^, respectively, under ambient conditions. MFM‐300(Al), while not achieving the highest gravimetric uptake, supersedes all aforementioned materials in terms of the packing density of NH_3_, 0.62 g cm^−3^ at 293 K. Interestingly, MFM‐300(Al) can liquefy NH_3_ above its boiling point (243 K) by reaching a density of 0.70 g cm^−3^ at 273 K, eliminating the need for energy intensive liquefaction for storage.

**Figure 1:**
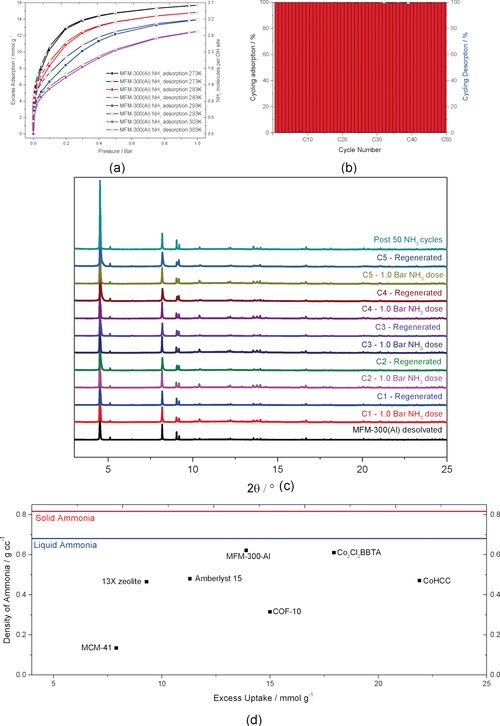
a) Adsorption isotherms of NH_3_ in MFM‐300(Al) at 273–303 K. b) Repeated cycling of NH_3_ up to 50 cycles in MFM‐300(Al) at 293 K with percentage adsorption (red) as a function of cycle number and the corresponding desorption (blue) reached for each cycle under pressure‐swing conditions. c) PXRD of in MFM‐300(Al) cycling with NH_3_. d) Comparison of densities of stored NH_3_ in MCM‐41,5a 13X zeolite,5a Amberlyst 15,5a MFM‐300(Al), COF‐10,^12^ Co_2_Cl_2_BBTA9c and CoHCC9a plotted against their respective gravimetric uptake compared with the density of liquid and solid NH_3_.

Significantly, adsorption of NH_3_ in MFM‐300(Al) is highly reversible with no loss of uptake capacity or crystallinity, and no broadening of Bragg peaks (Figure S4 in the Supporting Information) was observed after 50 adsorption–desorption cycles at 293 K (Figure 1). CoHCC is able to undergo four cycles with no loss of NH_3_ uptake; however, this necessitates reactivation at 150 °C under dynamic vacuum for 24 hrs.9a Furthermore, each isotherm adsorption point requires 170 minutes for equilibration, thus restricting its potential for portable NH_3_ storage. [Co_2_Cl_2_BBTA] and COF‐10 both show a loss of NH_3_ uptake after repeated cycling of 5.6 % and 4.5 %, respectively, and for complete regeneration both require heating to 200 °C under dynamic vacuum.9c, 12 In contrast, MFM‐300(Al) is able to repeatedly adsorb from regenerated MOF to complete saturation in 6 mins and completely desorb (saturation to full release) within ≈13.5 mins using a standard pressure‐swing over 50 cycles, making it an ideal candidate for NH_3_ storage.

The binding domains for adsorbed NH_3_ molecules within MFM‐300(Al) have been elucidated by in situ neutron powder diffraction (NPD). Structural analysis via Rietveld refinement of NPD data for 1.5 ND_3_/Al‐loaded MFM‐300(Al) identified three distinct binding sites (I, II and III) in [Al_2_(OH)_2_(L)](ND_3_)_3_ (Figure 2). Site I [occupancy=0.736(6)] is 1.76(2) Å {O → N1=2.84(1) Å} from the bridging μ_2_‐OH moiety in a hydrogen bonding pocket formed by the pore wall (Figure 2). The sub 2 Å distance and the high isosteric heat of adsorption (*Q*_st_>40 kJ mol^−1^) are indicative of a strong binding mode being present between NH_3_ and MFM‐300(Al).^13^ Sites II [occupancy=0.236(3)] and III [occupancy=0.213(5)] lie at a distance of 2.68(1) Å {N1 → N2=3.63(1) Å} and 2.29(3) Å {N2 → N3=3.09(1) Å} from site I and site II, respectively. This cooperative network of ND_3_ molecules propagates down the length of the 1D channel, anchored in place by site I. Bond distances for sites II⋅⋅⋅III and a slightly lengthened site I⋅⋅⋅II are similar to a typical inter‐molecular bond between ND_3_ molecules in the solid state at 2 K [N⋅⋅⋅D=2.357(2) Å],^14^ whereas the bond between the framework μ_2_‐OH and site I is significantly shorter. The structure for ND_3_‐loaded MFM‐300(Al) has also been determined at a loading of 0.5 ND_3_/Al and 1.0ND_3_/Al and these have shown similar binding sites as discussed above. With increased loading from 0.5 ND_3_/Al to 1.5 ND_3_/Al, we observed an overall shortening of the framework μ_2_‐OH⋅⋅⋅site I and sites I⋅⋅⋅II and an increase in the site II⋅⋅⋅III.

**Figure 2:**
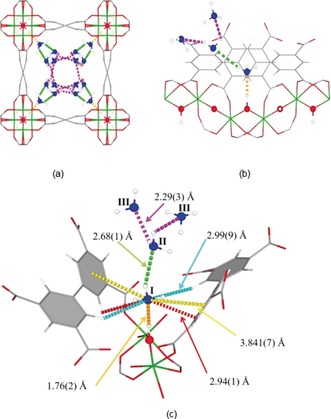
View of the structure of 1.5ND_3_/Al‐loaded MFM‐300(Al) determined by in situ NPD studies. a) View down the c‐axis; b) side on view of three binding sites in relation to the OH functionality; c) view of binding of ND_3_ to the framework. Framework hydrogen → Site I (orange)=1.76(2) Å {O → N1=2.84(1) Å}. Linker ring → Site I (yellow)=3.841(7) Å. Linker H1 → Site I (red)=2.94(1) Å. Linker H2 → Site I (indigo)=2.994(9) Å. Site I → Site II (green)=2.68(1) Å {N1 → N2=3.63(1) Å}. Site II → Site III (purple)=2.29(3) Å {N2 → N3=3.09(1) Å}.

Refinement of the NPD data for ND_3_‐loaded MFM‐300(Al) revealed an interesting observation: as the loading of ND_3_/μ_2_‐OH was increased, the hydrogen on hydroxyl groups underwent a reversible site exchange with the deuterium from guest ND_3_ molecules residing at Site I in the pore. This exchange is very distinct in the analysis of NPD data owing to the significant difference on neutron scattering of hydrogen and deuterium. We noted as the loading of ND_3_ increased from 0 to 0.5 ND_3_/μ_2_‐OH, the occupancy of the hydrogen of the μ_2_‐OH group decreased from 1.0 to 0.794(7) (Table S6). As the loading of ND_3_ was further increased to 1.0 and 1.5 ND_3_/μ_2_‐OH, the occupancy of the hydrogen on the μ_2_‐OH group decreased to 0.416(10) and the site was replaced by a deuterium to give a μ_2_‐OD [D occupancy=0.468(9) and 0.584(10), respectively].

We sought to examine the reversibility of this H–D exchange via in situ synchrotron FTIR micro‐spectroscopy by monitoring the νO‐H stretching vibration at 3692 cm^−1^ as a function of ND_3_ loading at 293 K (Figure 3). A rapid depletion of this band was observed on adsorption of ND_3_ under flow conditions, accompanied by the growth of a new band at 2720 cm^−1^ assigned to the νO‐D stretching mode, thus confirming H→D exchange. Once the H→D exchange is completed to give [Al_2_(OD)_2_(L)] with no residual O‐H stretching band observed, the material was charged with a flow of NH_3_ at 293 K. Interestingly, the νO‐D stretching band at 2720 cm^−1^ disappeared and the νO‐H band at 3692 cm^−1^ returned, indicating that the H→D exchange is completely reversible. It is worth noting that such H–D reversible exchange does not lead to any detectable structural degradation of the long range order of the framework (Figure S8). Traditionally, chemisorption and physisorption is distinguished on the basis of host–guest binding interaction and the formation of adsorbate–adsorbent bonds at the interface. Significantly, the adsorption of ND_3_ in MFM‐300(Al) revealed a new type of adsorption where adsorbent and adsorbates undergo rapid site‐exchange via reversible formation and cleavage of O−H and O−D chemical bonds.

**Figure 3:**
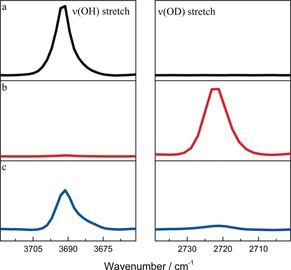
Reversible switching of framework hydroxyl hydrogen from H→D→H. The ν(OH) stretching vibration is at 3692 cm^−1^ and the ν(OD) stretching vibration at 2720 cm^−1^. a) Bare MFM‐300(Al) (black); b) ND_3_ exposed MFM‐300(Al) (red) and c) regenerated hydroxyl H functionalised MFM‐300(Al) after exposure of (b) to NH_3_ (blue).

In summary, MFM‐300(Al) shows excellent NH_3_ adsorption capacity with the intrinsic ability to achieve liquefaction of NH_3_ under near ambient conditions, and outperforms the state‐of‐the‐art porous materials in terms of NH_3_ packing density, reversibility and stability. MFM‐300(Al) offers unparalleled repeatable uptake characteristics and, coupled with the pseudo‐chemisorption binding mechanism, is a promising NH_3_ storage material for portable applications.

CCDC 1856081, 1856082, 1856083 and 1856084 contain the supplementary crystallographic data for this paper. These data can be obtained free of charge from The Cambridge Crystallographic Data Centre. Correspondence and requests for materials should be addressed to S.Y. (Sihai.Yang@manchester.ac.uk) and M.S. (M.Schroder@manchester.ac.uk).

## Conflict of interest

The authors declare no conflict of interest.

## Supporting information

As a service to our authors and readers, this journal provides supporting information supplied by the authors. Such materials are peer reviewed and may be re‐organized for online delivery, but are not copy‐edited or typeset. Technical support issues arising from supporting information (other than missing files) should be addressed to the authors.

Supplementary
