# Supplementary material for: Ammonia Storage by Reversible Host–Guest Site Exchange in a Robust Metal–Organic Framework
Source: Angew Chem Int Ed Engl. 2018 Oct 1;57(45):14778–81. doi: 10.1002/anie.201808316 (PMC6391960; doi:10.1002/anie.201808316)
Supplement: Supplementary file 1 — Supplementary [file ANIE-57-14778-s001.pdf]

Angewandte Chemie
Eine Zeitschrift der Gesellschaft Deutscher Chemiker

Supporting Information

# Ammonia Storage by Reversible Host–Guest Site Exchange in a Robust Metal–Organic Framework

*Harry G. W. Godfrey, Ivan da Silva, Lydia Briggs, Joseph H. Carter, Christopher G. Morris, Mathew Savage, Timothy L. Easun, Pascal Manuel, Claire A. Murray, Chiu C. Tang, Mark D. Frogley, Gianfelice Cinque, Sihai Yang,* and Martin Schröder**

anie_201808316_sm_miscellaneous_information.pdf

# Supporting Information

## 1. Experimental Section

### 1.1 Synthesis of MFM-300(Al)

Synthesis of $[Al_2(OH)_2(C_{16}O_8H_6)](H_2O)_6$ (MFM-300(Al)-solvate) and activation to give $[Al_2(OH)_2(C_{16}O_8H_6)]$ MFM-300(Al) were carried out using our previously reported method.[1]

### 1.2 Ammonia Safety

All systems involved in the supply, delivery and measurement of $NH_3$ were rigorously leak tested and used only within range of a Crowcon Gasman Portable Detector with a sensitivity of 1 ppm. All gases were exhausted from the experimental setup using a Nederman© BenchTop Extraction Arm.

### 1.3 Gas Adsorption and Cycling

Measurements of static adsorption isotherms (0 – 1.0 Bar) for $NH_3$ were carried out using an Xemis Gravimetric Sorption Analyser (Hiden Isochema, Warrington, UK). Desolvated samples of MFM-300(Al) were generated *in situ* under dynamic vacuum ($1x10^{-8}$ mbar) at 200°C for 24hrs. Research grade $NH_3$ was purchased from BOC and used as received. The pressure of $NH_3$ was increased from vacuum ($1x10^{-8}$ mbar) to 500 mbar and the uptake recorded. The pressure was then reduced to regenerate the sample with no assisted heating. This cycling process was repeated 50 times.

### 1.4 *In situ* Neutron Powder Diffraction (NPD)

Structural determination of the binding position of $ND_3$ within MFM-300(Al) was conducted using WISH, a long wavelength powder and single crystal neutron diffractometer at the ISIS neutron and muon facility at Rutherford Appleton Laboratory (UK). The instrument views a solid methane moderator providing a high flux of cold neutrons with a large bandwidth, transported to the sample *via* an elliptical guide. The WISH divergence jaws system allows tuning of the resolution according to the need of the experiment; in this case, it was setup in high resolution mode. The WISH detectors are 1m long, 8mm diameter pixelated $^3He$ tubes positioned at

2.2m from the sample and arranged on a cylindrical locus covering a 2θ scattering angle of 10-170°. To reduce the background from the sample environment, WISH was equipped with an oscillating radial collimator that defines a cylinder of radius of approximately 22 mm diameter at 90 ° scattering.[2]

The sample of desolvated MFM-300(Al) was loaded into a cylindrical vanadium sample container with an indium vacuum seal connected to a gas handling system. The sample was degassed at $1x10^{-7}$ mbar and at 100 °C for 4 days with regular helium flushing to remove any remaining trace guest water molecules. The sample was dosed with $ND_3$ using the volumetric method after being heated to room temperature to ensure that the gas is well dispersed throughout the crystalline structure of MFM-300(Al). Data collection for desolvated MFM-300(Al) and three subsequent loadings of $ND_3$ (0.5, 1.0 and 1.5 $ND_3$ molecules per OH functionality) were performed controlled using a helium cryostat (10 ± 0.2 K).

**1.5 *In Situ* High Resolution Synchrotron X-ray Powder Diffraction (PXRD)**

*In situ* high resolution powder X-ray diffraction experiments were conducted on beamline I11 Diamond Light Source (DLS), Rutherford Appleton Laboratories (UK). The I11 experimental set up involved a high brightness monochromatic beam being produced by a double-bounce Si(111) monochromator and harmonic rejection mirrors. The beam was delivered to the main instrument hutch where five multi-analysing crystal-detectors (MAC) travel in an arc around the sample.[3] Measurements were carried out in capillary mode and sample environment was controlled using an Oxford Cryosystems open-flow $N_2$ gas cryostat.

The sample was prepared by air drying a solvated MFM-300(Al) sample previously stored in acetone. The sample was then ground to provide a uniform particle size, packed into a borosilicate capillary (ø 0.7mm) and mounted into a diamond gas cell. MFM-300(Al) was desolvated under dynamic vacuum ($1 \times 10^{-6}$ mbar) for 16 hrs at 393 K. All collections and gas loadings were undertaken at 273 K. Gas loading of the MFM-300(Al) sample was achieved using a gas panel, the procedure for which was to reach the target pressure in the gas panel then expose the MOF sample to the gas. To ensure a state of equilibrium was reached, scans were performed to assess when changes in the diffraction pattern were no longer observable.

### 1.6 Synchrotron Infrared Micro-spectroscopy

Infrared micro-spectroscopy experiments were carried out using the B22: Multimode Infra-Red Imaging and Microspectroscopy (MIRIAM) beam line at the Diamond Light Source, Rutherford Appleton Laboratories (UK). The instrument is comprised of a Bruker Hyperion 3000 microscope in transmission mode, with a 15x objective and liquid $N_2$ cooled MCT detector, coupled to a Bruker Vertex 80 V Fourier Transform IR interferometer using radiation generated from a bending magnet source. Spectra were collected (512 scans) in the range 500-4000 $cm^{-1}$ at 4 $cm^{-1}$ resolution and an infrared spot size at the sample of approximately 20 × 20 μm. A microcrystalline powder of MFM-300(Al) was placed onto a S9 ZnSe disk and placed within a Linkam FTIR 600 gas-tight sample cell equipped with ZnSe windows, a heating stage and gas inlet and outlets. The $N_2$, $NH_3$ and $ND_3$ were pre-dried using individual zeolite filters. The analysis gases were dosed volumetrically to the sample cell using mass flow controllers, the total flow rate being maintained at 100 $cm^3$ $min^{-1}$ for all experiments. The gases were directly vented to an exhaust system and the total pressure in the cell was therefore 1 bar for all experiments. The sample was desolvated under a flow of dry $N_2$ at 100 $cm^3$ $min^{-1}$ and 393 K for 3 h. The sample was then cooled to 293 K under a continuous flow of $N_2$. Dry $NH_3$ was then dosed as a function of partial pressure, maintaining a total flow of 100 $cm^3$ $min^{-1}$. The sample was then regenerated with a flow of dry $N_2$. To investigate the H→D exchange reaction, a flow of $ND_3$ was introduced within the cell at a flow rate of 100 $cm^3$ $min^{-1}$ at 293K for 1hr. $N_2$ flushing was then repeated at 100 $cm^3$ $min^{-1}$, a scan taken and D→H exchange was implemented with a flow of $NH_3$ at 100 $cm^3$ $min^{-1}$. The spectrum was then observed after a final $N_2$ flushing at 100 $cm^3$ $min^{-1}$.

## 2. Additional Analysis of Gas Adsorption in MFM-300(Al)

### 2.1 Isoteric heat of adsorption for $NH_3$ in MFM-300(Al)

To calculate the differential enthalpies *($\Delta H_n$)* and entropies *($\Delta S_n$)* for $NH_3$ uptake as a function of loading *(n)*, all isotherms (273K – 303K) were fitted to the van't Hoff isochore (Equation 1).

$$\ln(p)_n = \frac{\Delta H_n}{RT} - \frac{\Delta S_n}{R} \quad (1)$$

A graph of *ln(p)* versus *1/T* at constant loading allows the differential enthalpy and entropy of adsorption and also the isosteric enthalpy of adsorption ($Q_{st,n}$) to be determined. Four example fittings are displayed in Figure S1. The calculated $R^2$ value for each fitting is > 0.99 indicating a reliable fit.

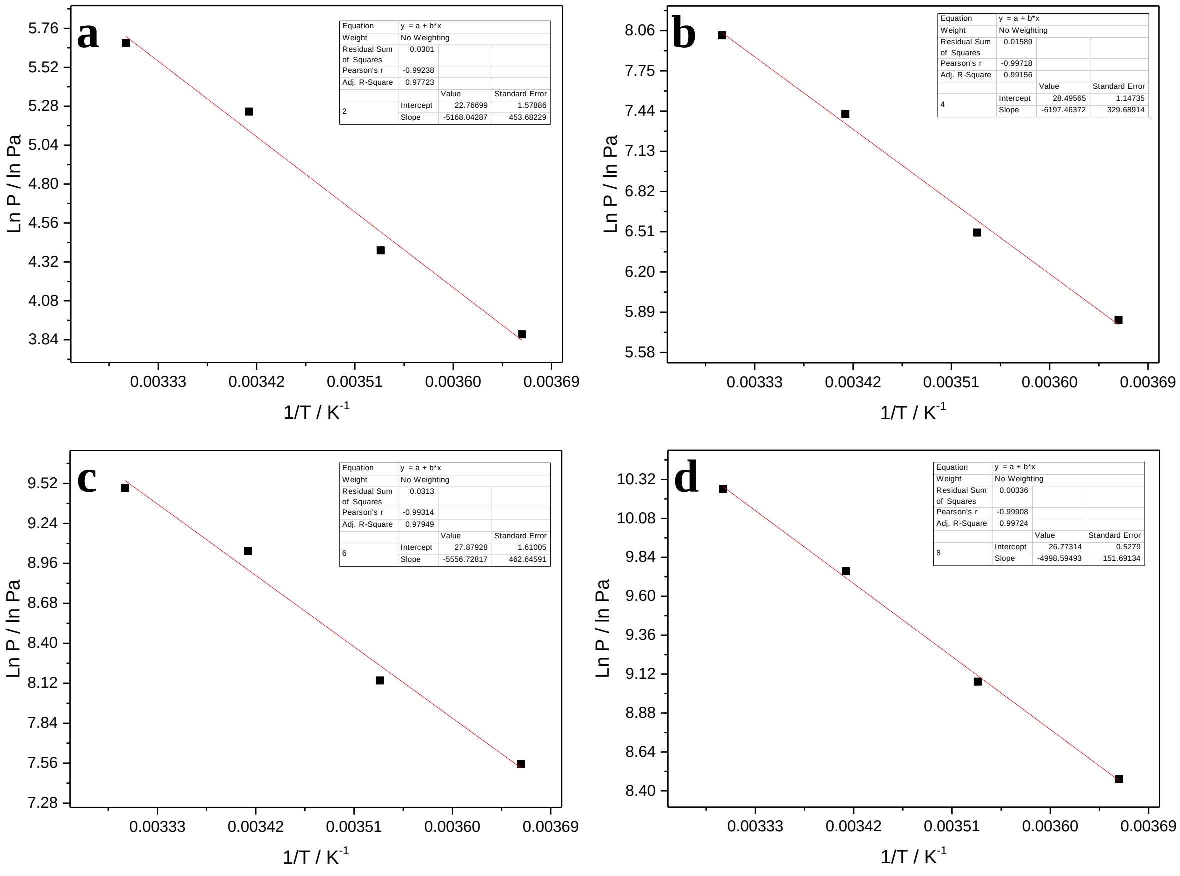


**Figure S1i:** Linear fittings of Van't Hoff isochore plots for adsorption isotherms of $NH_3$ in MFM-300(Al) at 2.0 (a), 4.0 (b), 6.0 (c) and 8.0 (d) mmol $g^{-1}$ loadings.

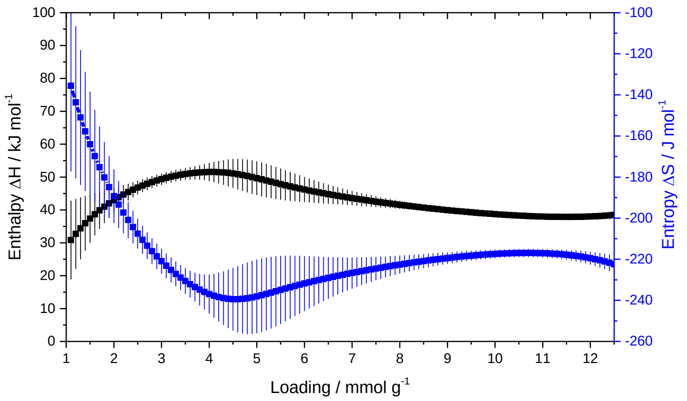


**Figure S1ii:** Isoteric heat of adsorption and entropy of adsorption calculated using the van't Hoff isochore for $NH_3$ adsorption in MFM-300(Al).

## 2.2 Dual-site Langmuir-Freundlich fittings and IAST selectivity of $NH_3$ vs $CO_2$, $CH_4$ and $N_2$.

Adsorption isotherms of $NH_3$, $CO_2$, $N_2$ and $CH_4$ in MFM-300(Al) at 293K were fitted with the dual-site Langmuir-Freundlich model (Equation 2), where $n$ is the loading in mmol $g^{-1}$, $P$ is the pressure in bar, $q_{sat1}$ is the saturation capacity in mmol $g^{-1}$, $b_1$ is the Langmuir parameter in $bar^{-1}$, and $v_1$ is the Freundlich parameter for two sites 1 and 2. All $R^2$ values for the fits are >0.999 confirming they fit the model well.

$$n = \frac{q_{sat1} b_1 P^{v_1}}{1 + b_1 P^{v_1}} + \frac{q_{sat2} b_2 P^{v_2}}{1 + b_2 P^{v_2}} \quad (2)$$

Ideal adsorbed solution theory (IAST)[4] was used to determine the selectivity factor, $S$, for binary mixtures using pure component isotherm data. The selectivity factor, $S$, is where $x_i$ is the amount of each component adsorbed as determined from IAST and $y_i$ is the mole fraction of each component in the gas phase at equilibrium (Equation 3). The IAST adsorption selectivities were calculated for a range of molar ratios of binary mixtures of $NH_3/CO_2$, $NH_3/CH_4$ and $NH_3/N_2$ at 293 K and at a pressure range of 0.1 - 1 bar (Figure S2).

$$S = \frac{x_1/y_1}{x_2/y_2} \quad (3)$$

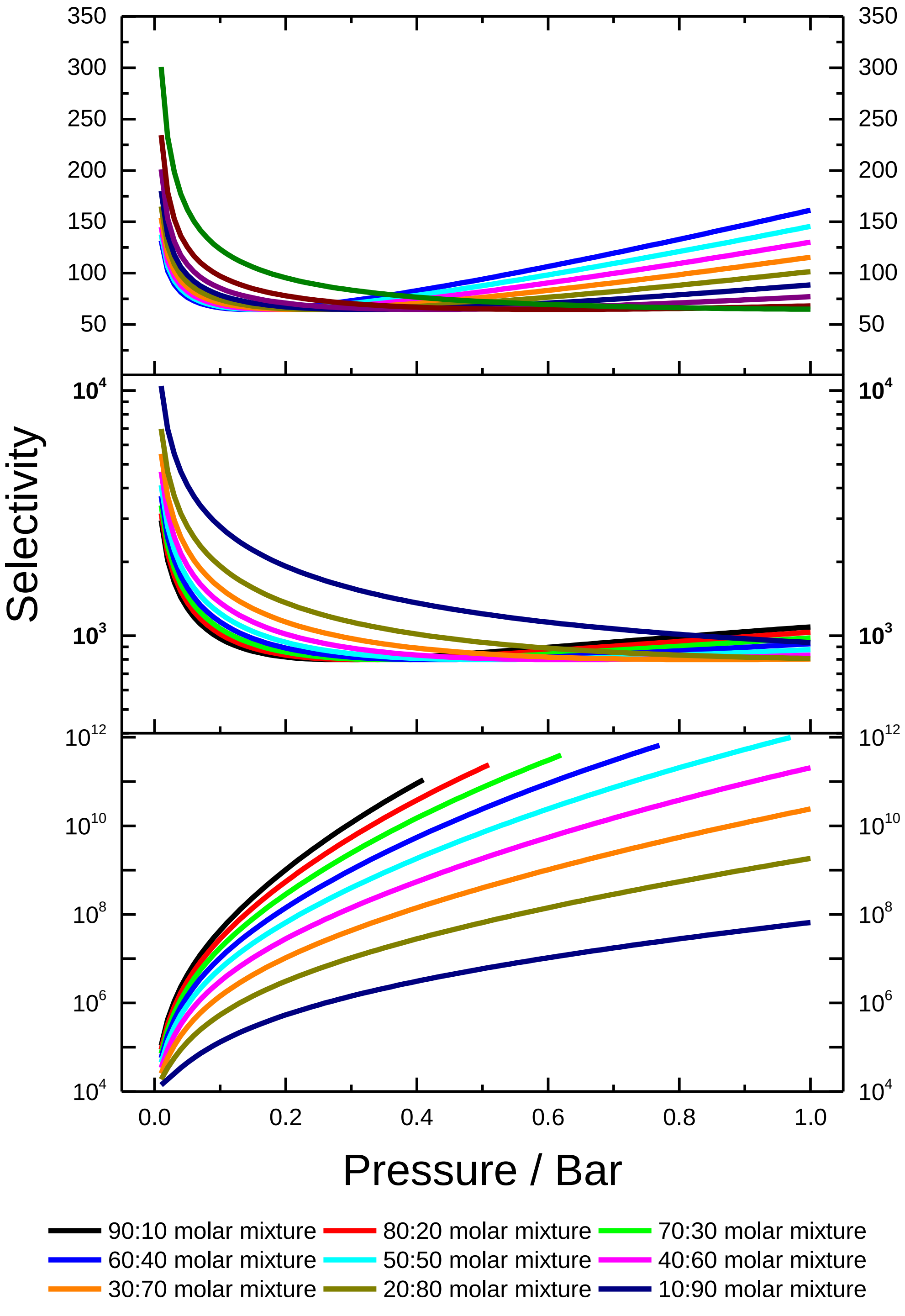


**Figure S2:** Selectivity calculated using the IAST method between $NH_3/CO_2$ (Top), $NH_3/N_2$ (Middle) and $NH_3/CH_4$ (Bottom) in 90:10 (Black), 80:20 (Red), 70:30 (Green), 60:40 (Blue), 50:50 (Cyan), 40:60 (Magenta), 30:70 (Orange), 20:80 (Dark Yellow) and 10:90 (Navy) molar ratios of each respective gas mixture.

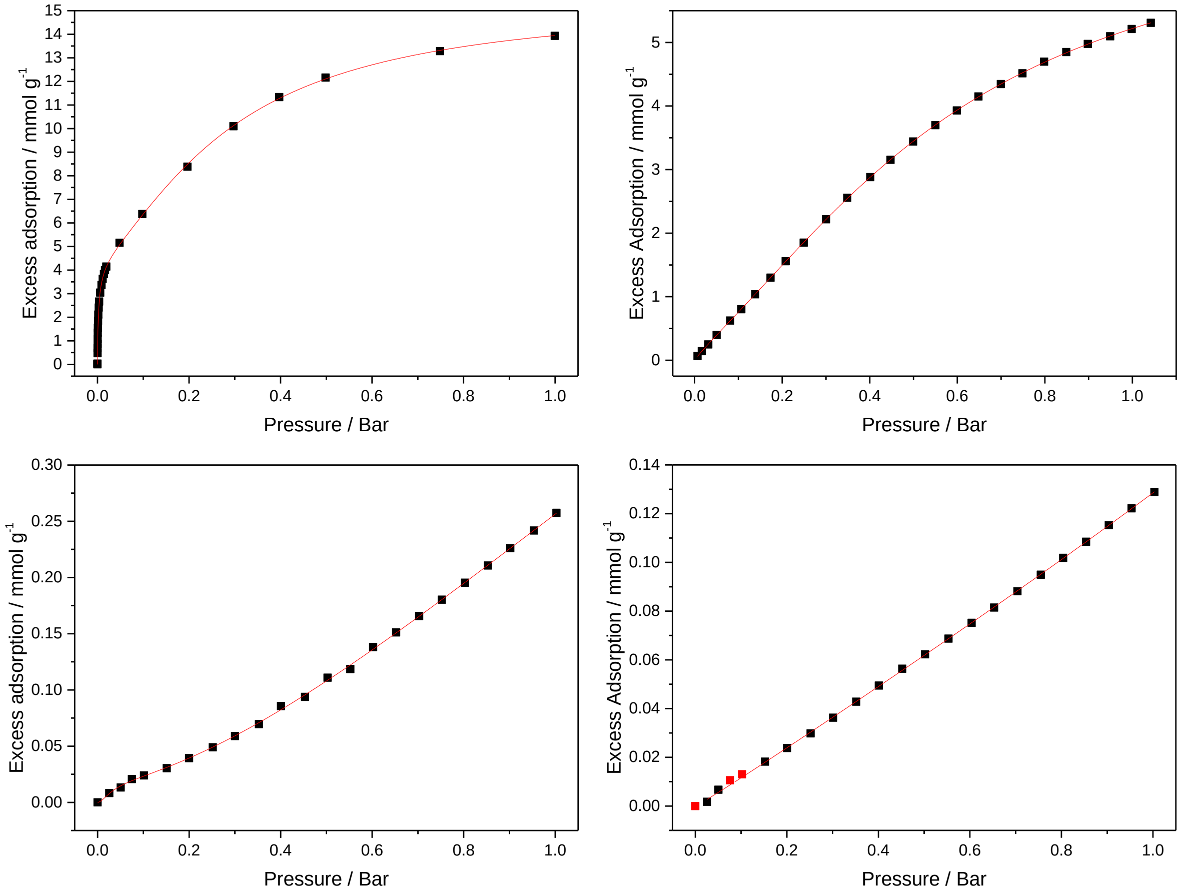


**Figure S3:** Adsorption of $NH_3$ (Top Right), $CO_2$ (Top Right), $CH_4$ (Bottom Left) and $N_2$ (Bottom Right) at 293K in MFM-300(Al) fitted using the dual-site Langmuir-Freundlich model (Red).

### 2.3 Comparative density of $NH_3$ in porous materials

**Table S1:** Summary of highest performing porous materials in the literature.

| Material | Material Type | BET surface Area / $m^2\ g^{-1}$ | Pore Volume / $cm^3\ Å^{-1}\ g^{-1}$ | Capacity / mmol $g^{-1}$ | Ammonia Packing Density / g $cc^{-1}$ | Cycling Ability | Regeneration | Notes | Reference |
|---|---|---|---|---|---|---|---|---|---|
| **CoHCC** | Porous Dye | 848 | 0.79[a] | 21.9 | 0.471 | 4 cycles, No loss | 150°C and dynamic vacuum 24hr (does not reach zero) | 2.8hrs per adsorption point | [5] |
| **$Co_2Cl_2$BBTA** | Metal Organic Framework | 1161 | 0.50[a] | 18.0 | 0.610 | 3 cycles, 5.6% loss | 200°C under dynamic vacuum | PXRD shows loss of long range order | [6] |
| **COF-10** | Covalent Organic Framework | 1200 | 0.81[a] | 15.0 | 0.315 | 3 cycles, 4.5% loss | 200°C for 12hr at high vacuum | Loss of surface area | [7] |
| **MFM-300(Al)** | Metal Organic Framework | 1325 | 0.38 | 13.9 | 0.622 | 50 cycles, No loss | Dynamic vacuum <1hr | - | This work |
| **Amberlyst 15** | Ion-exchange Resin | 225 | 0.40[a] | 11.3 | 0.480 | -* | -* | - | [8] |
| **13X zeolite** | Zeolite | 615 | 0.34[a] | 9.30 | 0.465 | -* | -* | - | [8] |
| **MCM-41** | Mesoporous Silica | 990 | 1.00 | 7.90 | 0.134 | -* | -* | - | [8] |

*- data not available. [a] – values estimated from published $N_2$ isotherms at 77K.

## 3. Crystallographic Analysis of Adsorbed Ammonia in MFM-300(Al)

### 3.1 Ammonia cycling stability determined by *in situ* high-res PXRD

Five separate cycles of dosing and removal of $NH_3$ in MFM-300(Al) were studied at beamline I11, DLS. This was to determine the crystallographic stability of the material to repeated exposure to $NH_3$ and whether it had any detrimental structural impact. Figure 1 shows the normalised diffraction patterns for this experiment and we observe no significant structural changes in MFM-300(Al) over 5 repeated cycles of $NH_3$.

To further ratify this, a full width at half maximim (FWHM) analysis on the (110), (211) and (112) peaks was undertaken. These peaks were selected for their relative intensity within the diffraction pattern and the different direction each plane is oriented. No significant peak broadening is observed over 5 cycles indicating that $NH_3$ is not causing structural changes (Figure S4).

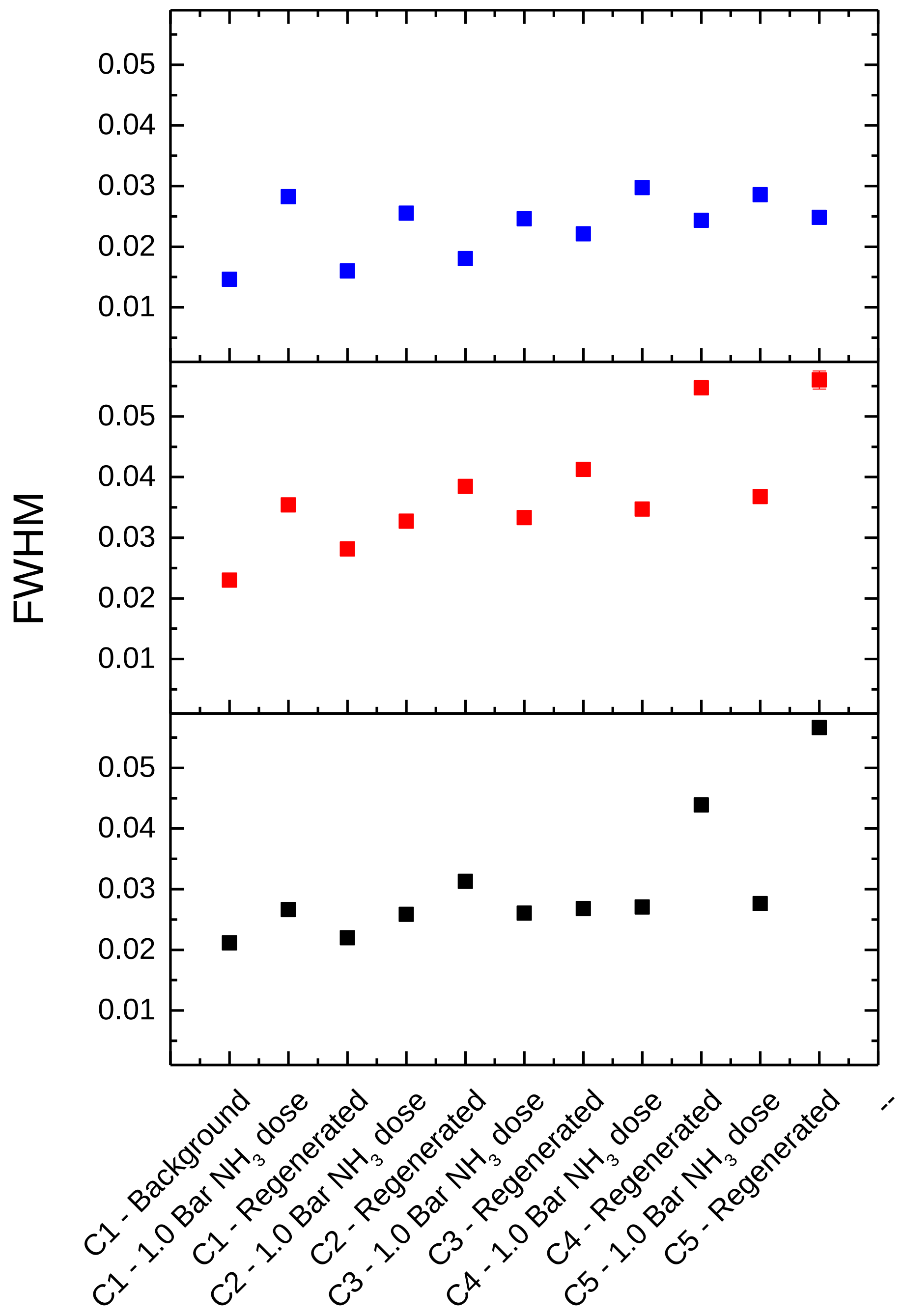


**Figure S4:** FWHM of (110) [Black], (211) [Red] and (112) [Blue] peaks as a function of repeated $NH_3$ loading and removal in MFM-300(Al) by high-resolution PXRD collected at beamline I11, DLS. Peaks fitted using a pseudo-Voigt function.

**Table S2:** Summary of the FWHM calculations for the (110), (211) and (112) peaks as a function of repeated $NH_3$ loading and their relative ratios to one another.

| | FWHM | | | Peak Ratios |
|---|---|---|---|---|
| | (110) peak | (211) peak | (112) peak | |
| **C1 - Background** | 0.02114 | 0.02301 | 0.01460 | 19:4:1 |
| **C1 - 1.0 Bar $NH_3$ dose** | 0.02663 | 0.03540 | 0.02823 | 21:5:1 |
| **C1 - Regenerated** | 0.02198 | 0.02813 | 0.01599 | 20:4:1 |
| **C2 - 1.0 Bar $NH_3$ dose** | 0.02584 | 0.03273 | 0.02552 | 18:4:1 |
| **C2 - Regenerated** | 0.03129 | 0.03843 | 0.01802 | 18:3:1 |
| **C3 - 1.0 Bar $NH_3$ dose** | 0.02603 | 0.03331 | 0.02461 | 22:5:1 |
| **C3 - Regenerated** | 0.02680 | 0.04126 | 0.02210 | 13:2:1 |
| **C4 - 1.0 Bar $NH_3$ dose** | 0.02707 | 0.03472 | 0.02973 | 15:4:1 |
| **C4 - Regenerated** | 0.04389 | 0.05472 | 0.02434 | 15:3:1 |
| **C5 - 1.0 Bar $NH_3$ dose** | 0.02762 | 0.03676 | 0.02857 | 14:3:1 |
| **C5 - Regenerated** | 0.05665 | 0.05601 | 0.02483 | 11:2:1 |

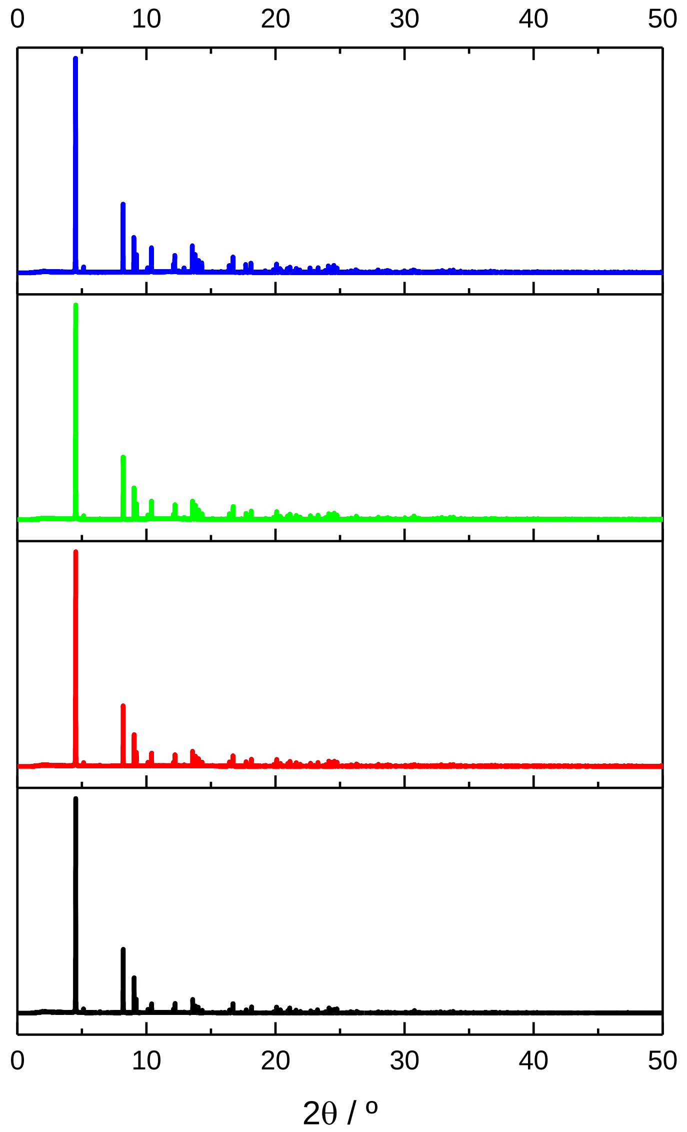


**Figure S5:** In situ high resolution powder X-ray diffraction of desolvated MFM-300(Al) (black), 0.04 bar $NH_3$-loaded MFM-300(Al) (red), 0.1 bar $NH_3$-loaded MFM-300(Al) (green) and 1 bar $NH_3$-loaded MFM-300(Al) (blue) collected at beamline I11, Diamond Light Source at 293K.

Figure S6 shows how the MFM-300(Al) crystallographic cell changes as a function of increased partial pressure of $NH_3$. We observe a small volume contraction, seen predominantly in the *c*-axis as the loading increases from 0 to approximately 0.1 bar. This is equivalent to 1.5 ammonia molecules being present per binding site. This cell contraction hints at a very strong interaction between the bridging $\mu_2$-OH and the guest $NH_3$ molecule. We

therefore propose that the first site is filling almost exclusively, indicated by the cell contraction. After this point, the pores fill to saturation causing an expansion in cell volume.

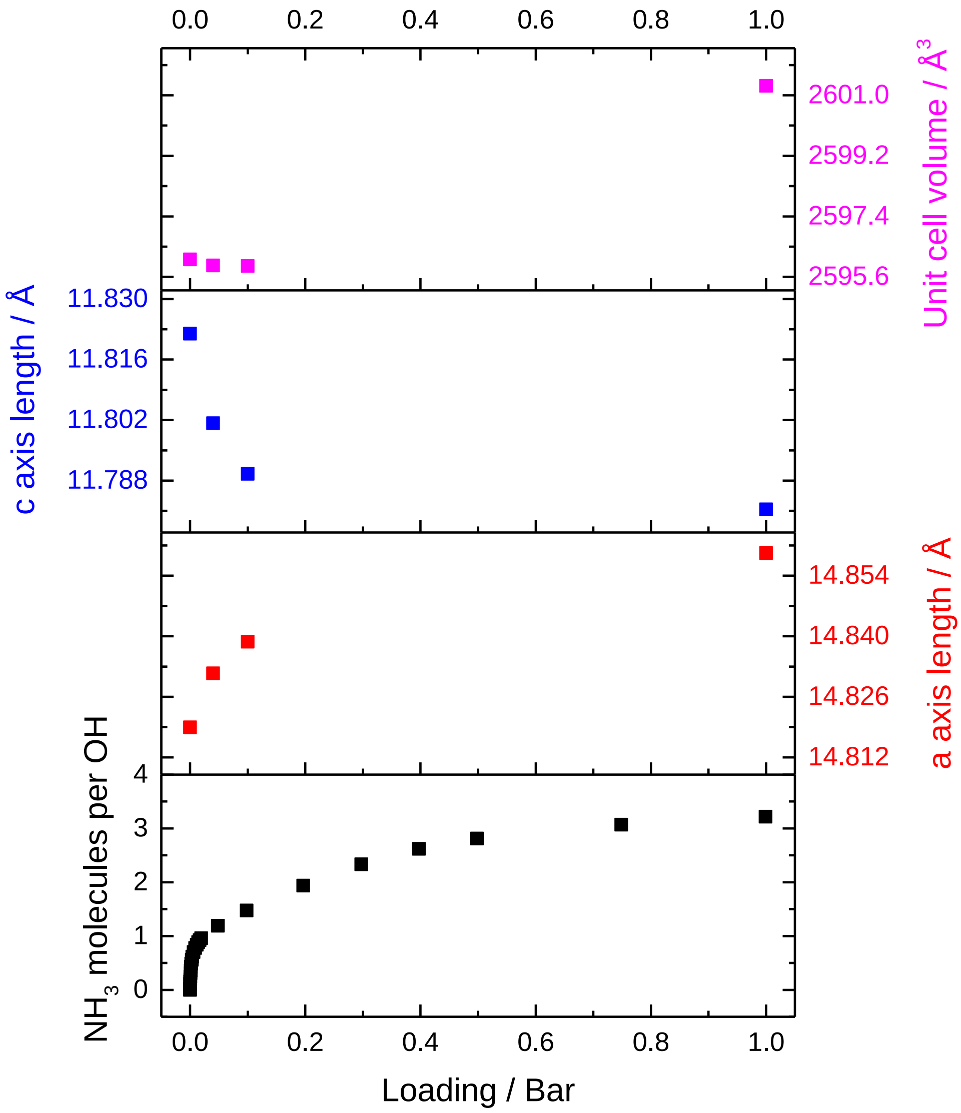


**Figure S6:** Comparison of unit cell parameters and $NH_3$ loading per OH functionality (black) in MFM-300(Al). Crystallographic *a* axis (red), crystallographic c axis (blue) and unit cell volume (magenta) calculated using a Pawley fitting with error bars displayed.

### 3.2 Direct visualisation of $NH_3$ in MFM-300(Al) by *in situ* high resolution NPD

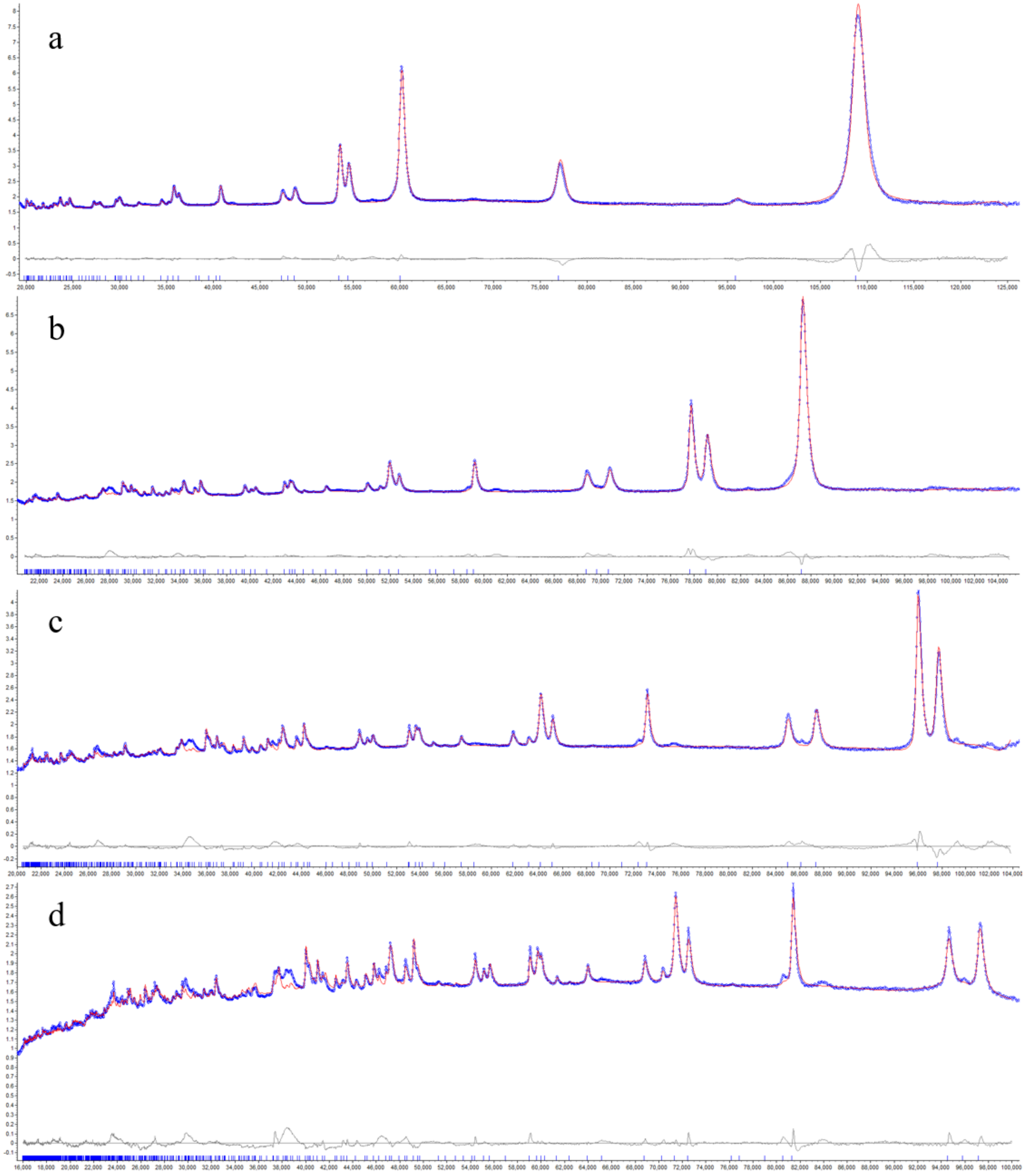


**Figure S7:** NPD diffraction patterns of desolvated MFM-300(Al) (blue = observed pattern, red = calculated pattern, grey = difference pattern, blue ticks = hkl ticks] from Bank 2 (a), Bank 3 (b), Bank 4 (c) and Bank 5 (d) collected at STFC-ISIS on the WISH diffractometer.

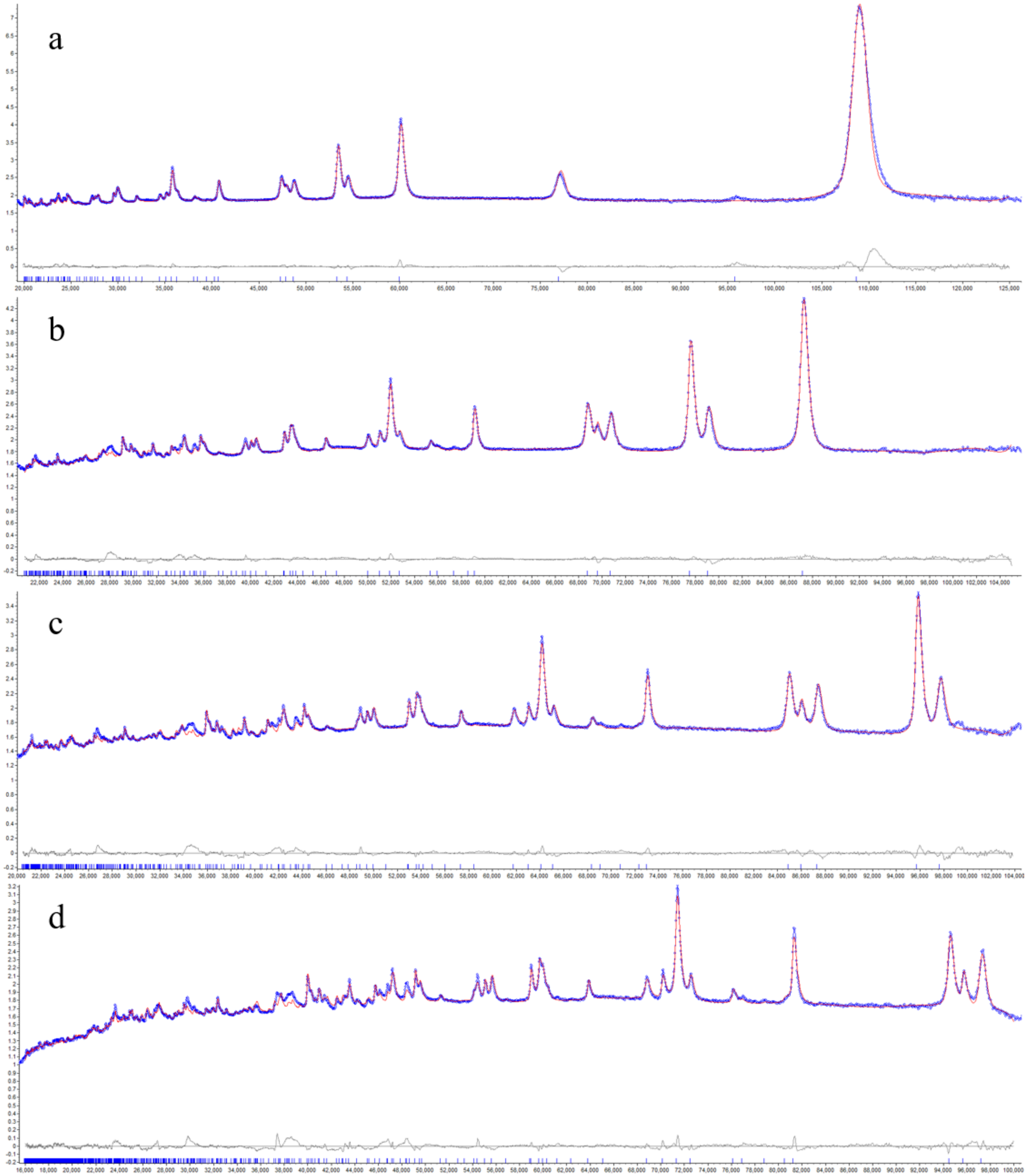


**Figure S8:** NPD diffraction patterns of MFM-300(Al)·$0.5ND_3$ (blue = observed pattern, red = calculated pattern, grey = difference pattern, blue ticks = hkl ticks] from Bank 2 (a), Bank 3 (b), Bank 4 (c) and Bank 5 (d) collected at STFC-ISIS on the WISH diffractometer.

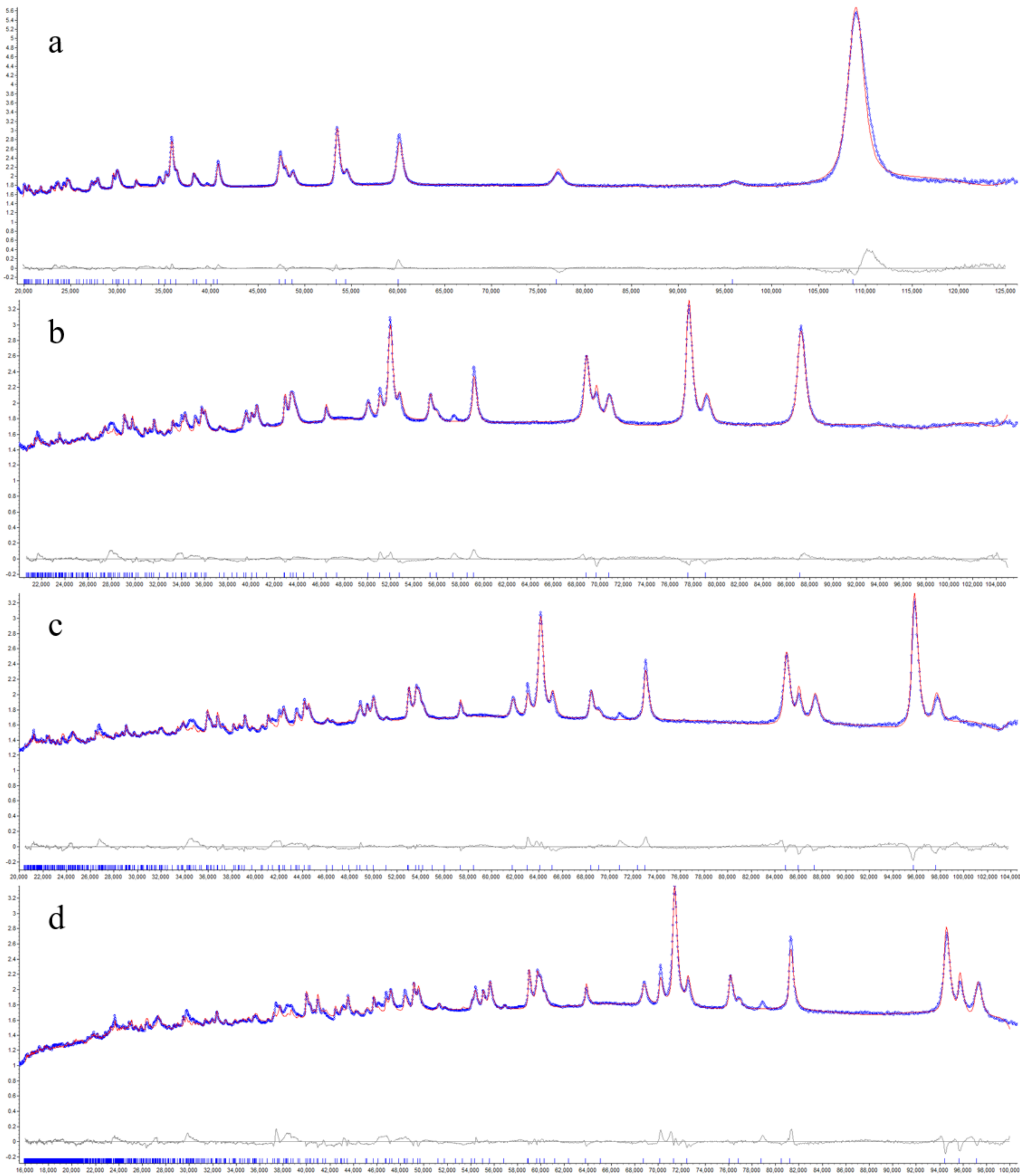

**Figure S9:** NPD diffraction patterns of MFM-300(Al)·1.0$ND_3$ (blue = observed pattern, red = calculated pattern, grey = difference pattern, blue ticks = hkl ticks] from Bank 2 (a), Bank 3 (b), Bank 4 (c) and Bank 5 (d) collected at STFC-ISIS on the WISH diffractometer.

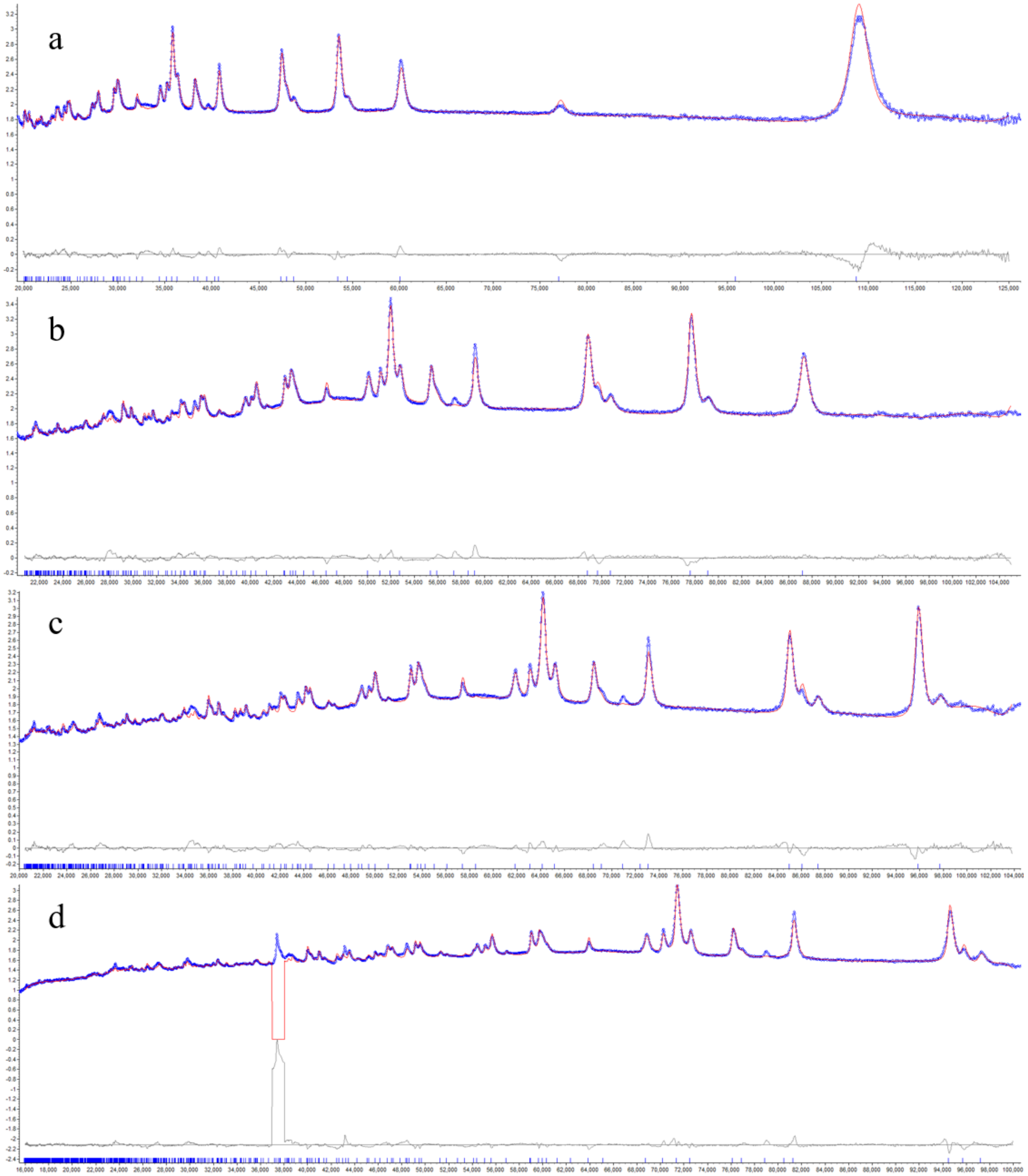


**Figure S10:** NPD diffraction patterns of MFM-300(Al)·1.5$ND_3$ (blue = observed pattern, red = calculated pattern, grey = difference pattern, blue ticks = hkl ticks] from Bank 2 (a), Bank 3 (b), Bank 4 (c) and Bank 5 (d) collected at STFC-ISIS on the WISH diffractometer.

**Table S3:** Atomic positions for atoms in MFM-300(Al)·0.5$ND_3$ ($R_{exp}$: 0.368133784, $R_{wp}$: 1.54349268, $R_p$: 1.10778478, GoF: 4.19274935)

| | ***x*** | ***y*** | ***z*** | ***Occupancy*** | ***$B_{iso}$ / Å²*** |
|---|---|---|---|---|---|
| Al | 0.6946(7) | 0.3054(7) | 0.5 | 1 | 0.27(10) |
| O1 | 0.7491(8) | 0.25 | 0.625 | 1 | 0.27(10) |
| O2 | 0.6244(10) | 0.3836(11) | 0.6047(9) | 1 | 0.27(10) |
| O3 | 0.6055(11) | 0.2844(7) | 0.7459(9) | 1 | 0.27(10) |
| C1 | 0.5939(8) | 0.3582(7) | 0.6978(6) | 1 | 0.27(10) |
| C2 | 0.5359(3) | 0.4251(2) | 0.7597(5) | 1 | 0.27(10) |
| C3 | 0.5 | 0.5 | 0.6994(6) | 1 | 0.27(10) |
| C4 | 0.5359(3) | 0.4251(2) | 0.8803(3) | 1 | 0.27(10) |
| C5 | 0.5 | 0.5 | 0.9406(3) | 1 | 0.27(10) |
| H3 | 0.5 | 0.5 | 0.6103(10) | 1 | 0.27(10) |
| H4 | 0.5624(6) | 0.3697(6) | 0.9249(5) | 1 | 0.27(10) |
| H1 | 0.822(3) | 0.25 | 0.625 | 0.794(7) | 0.33(12) |
| D1 | 0.822(3) | 0.25 | 0.625 | 0.206(7) | 0.33(12) |
| N1_1 | 0.748(3) | 0.629(3) | 0.554(4) | 0.065(3) | 0.7(4) |
| D1_1 | 0.769(6) | 0.578(5) | 0.605(7) | 0.065(3) | 0.7(4) |
| D2_1 | 0.756(7) | 0.687(4) | 0.599(8) | 0.065(3) | 0.7(4) |
| D3_1 | 0.681(3) | 0.620(6) | 0.544(9) | 0.065(3) | 0.7(4) |
| N1_2 | 0.625 | 0.75 | 0.625 | 0.108(4) | 0.7(4) |
| D1_2 | 0.571(3) | 0.767(5) | 0.576(5) | 0.054(2) | 0.7(4) |
| D2_2 | 0.680(3) | 0.751(7) | 0.574(6) | 0.054(2) | 0.7(4) |
| D3_2 | 0.616(5) | 0.685(2) | 0.648(8) | 0.054(2) | 0.7(4) |
| N1_4 | 0.75 | 0.0570(6) | 0.375 | 0.443(4) | 0.7(4) |
| D1_4 | 0.686719 | 0.0312(6) | 0.375 | 0.187(2) | 0.7(4) |
| D2_4 | 0.7816405 | 0.0312(6) | 0.3061069 | 0.187(2) | 0.7(4) |
| D3_4 | 0.7816405 | 0.0312(6) | 0.4438931 | 0.187(2) | 0.7(4) |
| H1_4 | 0.686719 | 0.0312(6) | 0.375 | 0.0343(12) | 0.7(4) |
| H2_4 | 0.7816405 | 0.0312(6) | 0.3061069 | 0.0343(12) | 0.7(4) |
| H3_4 | 0.7816405 | 0.0312(6) | 0.4438931 | 0.0343(12) | 0.7(4) |

**Table S4:** Atomic positions for atoms in MFM-300(Al)·0.909$ND_3$ ($R_{exp}$: 0.279228915, $R_{wp}$: 1.63039385, $R_p$: 1.17169904, GoF: 5.83891482)

| | ***x*** | ***y*** | ***z*** | ***Occupancy*** | ***$B_{iso}$ / Å²*** |
|---|---|---|---|---|---|
| Al | 0.6966(8) | 0.3034(8) | 0.5 | 1 | 0.37(11) |
| O1 | 0.7494(8) | 0.25 | 0.625 | 1 | 0.37(11) |
| O2 | 0.6248(10) | 0.3816(11) | 0.6010(8) | 1 | 0.37(11) |
| O3 | 0.6079(11) | 0.2844(7) | 0.7452(9) | 1 | 0.37(11) |
| C1 | 0.5957(8) | 0.3589(6) | 0.6968(6) | 1 | 0.37(11) |
| C2 | 0.5409(3) | 0.4277(2) | 0.7599(6) | 1 | 0.37(11) |
| C3 | 0.5 | 0.5 | 0.6996(7) | 1 | 0.37(11) |
| C4 | 0.5409(3) | 0.4277(2) | 0.8804(4) | 1 | 0.37(11) |
| C5 | 0.5 | 0.5 | 0.9406(4) | 1 | 0.37(11) |
| H3 | 0.5 | 0.5 | 0.6105(11) | 1 | 0.37(11) |
| H4 | 0.5711(6) | 0.3743(6) | 0.9249(6) | 1 | 0.37(11) |
| H1 | 0.821(3) | 0.25 | 0.625 | 0.532(9) | 0.44(13) |
| D1 | 0.821(3) | 0.25 | 0.625 | 0.468(9) | 0.44(13) |
| N1_1 | 0.7358(17) | 0.5939(19) | 0.6546(16) | 0.137(3) | 1.7(3) |
| D1_1 | 0.6690(18) | 0.605(4) | 0.644(4) | 0.137(3) | 1.7(3) |
| D2_1 | 0.743(4) | 0.527(2) | 0.673(4) | 0.137(3) | 1.7(3) |
| D3_1 | 0.754(4) | 0.628(3) | 0.725(3) | 0.137(3) | 1.7(3) |
| N1_2 | 0.625 | 0.75 | 0.625 | 0.105(5) | 1.7(3) |
| D1_2 | 0.617(8) | 0.6822(10) | 0.629(14) | 0.053(2) | 1.7(3) |
| D2_2 | 0.686(3) | 0.764(8) | 0.659(8) | 0.053(2) | 1.7(3) |
| D3_2 | 0.578(4) | 0.777(9) | 0.678(7) | 0.053(2) | 1.7(3) |
| N1_4 | 0.75 | 0.0529(5) | 0.375 | 0.667(5) | 1.7(3) |
| D1_4 | 0.686732 | 0.0271(5) | 0.375 | 0.256(3) | 1.7(3) |
| D2_4 | 0.781634 | 0.0271(5) | 0.3061477 | 0.256(3) | 1.7(3) |
| D3_4 | 0.781634 | 0.0271(5) | 0.4438523 | 0.256(3) | 1.7(3) |
| H1_4 | 0.686732 | 0.0271(5) | 0.375 | 0.0780(15) | 1.7(3) |
| H2_4 | 0.781634 | 0.0271(5) | 0.3061477 | 0.0780(15) | 1.7(3) |
| H3_4 | 0.781634 | 0.0271(5) | 0.4438523 | 0.0780(15) | 1.7(3) |

**Table S5:** Atomic positions for atoms in MFM-300(Al)·1.185$ND_3$ ($R_{exp}$: 0.329338862, $R_{wp}$: 1.4893783, $R_p$: 1.08856582, GoF: 4.52232783)

| | ***x*** | ***y*** | ***z*** | ***Occupancy*** | ***$B_{iso}$ / Å²*** |
|---|---|---|---|---|---|
| Al | 0.6967(9) | 0.3033(9) | 0.5 | 1 | 1.76(14) |
| O1 | 0.7551(10) | 0.25 | 0.625 | 1 | 1.76(14) |
| O2 | 0.6243(11) | 0.3829(12) | 0.6079(9) | 1 | 1.76(14) |
| O3 | 0.6022(12) | 0.2867(8) | 0.7438(11) | 1 | 1.76(14) |
| C1 | 0.5946(9) | 0.3584(7) | 0.6973(7) | 1 | 1.76(14) |
| C2 | 0.5399(3) | 0.4273(2) | 0.7602(6) | 1 | 1.76(14) |
| C3 | 0.5 | 0.5 | 0.7001(7) | 1 | 1.76(14) |
| C4 | 0.5399(3) | 0.4273(2) | 0.8805(4) | 1 | 1.76(14) |
| C5 | 0.5 | 0.5 | 0.9407(4) | 1 | 1.76(14) |
| H3 | 0.5 | 0.5 | 0.6111(12) | 1 | 1.76(14) |
| H4 | 0.5694(6) | 0.3735(7) | 0.9250(6) | 1 | 1.76(14) |
| H1 | 0.828(2) | 0.25 | 0.625 | 0.416(10) | 2.11(17) |
| D1 | 0.828(2) | 0.25 | 0.625 | 0.584(10) | 2.11(17) |
| N1_1 | 0.7005(10) | 0.5796(8) | 0.6237(13) | 0.236(3) | 1.8(3) |
| D1_1 | 0.678(2) | 0.6128(15) | 0.5541(19) | 0.236(3) | 1.8(3) |
| D2_1 | 0.703(3) | 0.6254(15) | 0.687(2) | 0.236(3) | 1.8(3) |
| D3_1 | 0.7649(15) | 0.561(4) | 0.608(3) | 0.236(3) | 1.8(3) |
| N1_2 | 0.625 | 0.75 | 0.625 | 0.213(5) | 1.8(3) |
| D1_2 | 0.623(3) | 0.6838(14) | 0.646(7) | 0.106(2) | 1.8(3) |
| D2_2 | 0.615(3) | 0.785(5) | 0.698(4) | 0.106(2) | 1.8(3) |
| D3_2 | 0.570(2) | 0.762(4) | 0.575(4) | 0.106(2) | 1.8(3) |
| N1_4 | 0.75 | 0.0529(5) | 0.375 | 0.736(6) | 1.8(3) |
| D1_4 | 0.686766 | 0.0271(5) | 0.375 | 0.271(3) | 1.8(3) |
| D2_4 | 0.781617 | 0.0271(5) | 0.306213 | 0.271(3) | 1.8(3) |
| D3_4 | 0.781617 | 0.0271(5) | 0.443787 | 0.271(3) | 1.8(3) |
| H1_4 | 0.686766 | 0.0271(5) | 0.375 | 0.0974(16) | 1.8(3) |
| H2_4 | 0.781617 | 0.0271(5) | 0.306213 | 0.0974(16) | 1.8(3) |
| H3_4 | 0.781617 | 0.0271(5) | 0.443787 | 0.0974(16) | 1.8(3) |

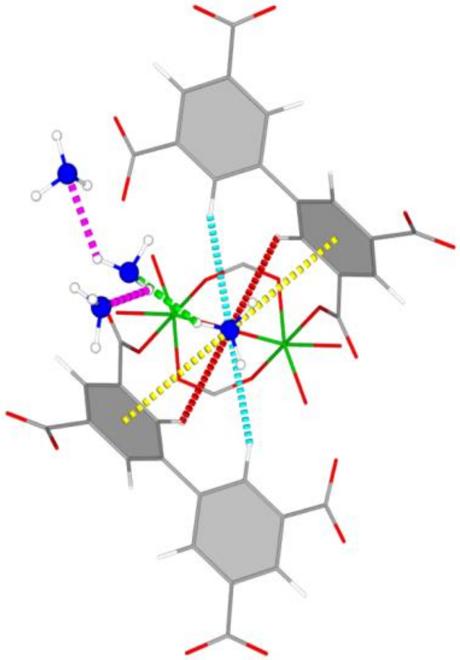

**Figure S11:** View of the active binding site determined *via* NPD data of the MFM-300(Al)·1.5$ND_3$

**Table S6:** Summary of NPD binding models

<table>
<tr><th></th><th colspan="6">0.5 $ND_3$ per hydroxyl</th><th colspan="6">1.0 $ND_3$ per hydroxyl</th><th colspan="6">1.5 $ND_3$ per hydroxyl</th></tr>
<tr><td>Framework OH······Site I (Orange)<br>{O······N distance}</td><td colspan="6">1.79(4) Å<br>{2.871(9) Å}</td><td colspan="6">1.86(4) Å<br>{2.92(1) Å}</td><td colspan="6">1.76(2) Å<br>{2.84(1) Å}</td></tr>
<tr><td>Site I······Site II (Green)<br>{N······N distance}</td><td colspan="6">3.83(4) Å<br>{4.67(4) Å}</td><td colspan="6">2.54(2) Å<br>{3.39(2) Å}</td><td colspan="6">2.68(1) Å<br>{3.63(1) Å}</td></tr>
<tr><td>Site II······Site III (Purple)<br>{N······N distance}</td><td colspan="6">2.17(7) Å<br>{2.68(4) Å}</td><td colspan="6">2.25(3) Å<br>{2.85(2) Å}</td><td colspan="6">2.29(3) Å<br>{3.09(1) Å}</td></tr>
<tr><td>Occupancy of framework Hydrogen/ Deuterium</td><td colspan="3">0.794(7)</td><td colspan="3">0.206(7)</td><td colspan="3">0.532(9)</td><td colspan="3">0.468(9)</td><td colspan="3">0.416(10)</td><td colspan="3">0.584(10)</td></tr>
<tr><td>Occupancy of Site I/ Site II/ Site III</td><td colspan="2">0.443(4)</td><td colspan="2">0.108(4)</td><td colspan="2">0.065(3)</td><td colspan="2">0.667(5)</td><td colspan="2">0.137(3)</td><td colspan="2">0.105(5)</td><td colspan="2">0.736(6)</td><td colspan="2">0.236(3)</td><td colspan="2">0.213(5)</td></tr>
</table>

**4. Binding of $NH_3$ Studied *by in situ* FTIR**

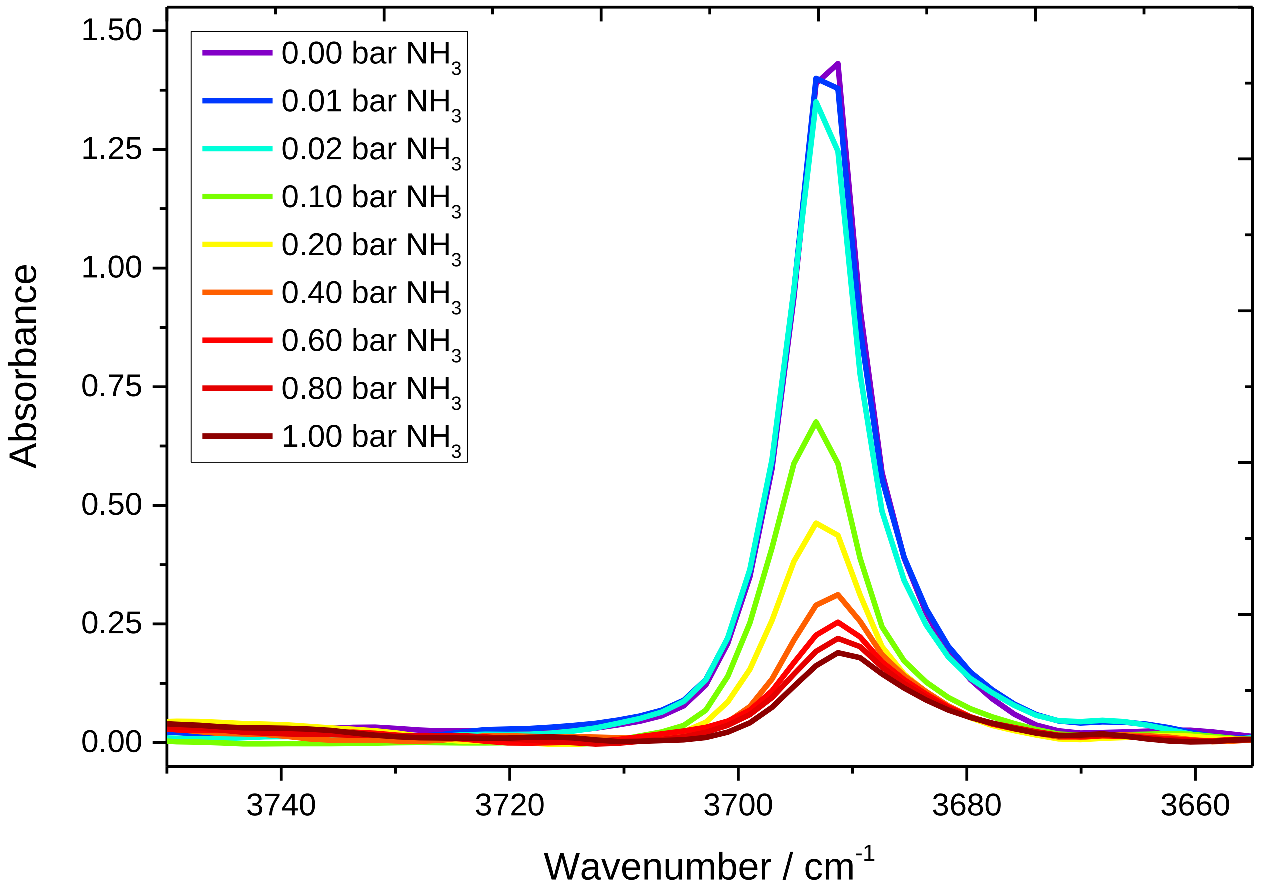


**Figure S14:** Stepwise loading of $NH_3$ in MFM-300(Al) at partial pressures ranging from 0-1 bar. Peak position of bare ν(OH) stretching vibration at 3691.60 $cm^{-1}$ decreases as a function of $NH_3$ loading (Maroon). Small peak at 3685.95 $cm^{-1}$ shifts as a function of $NH_3$ partial pressure.

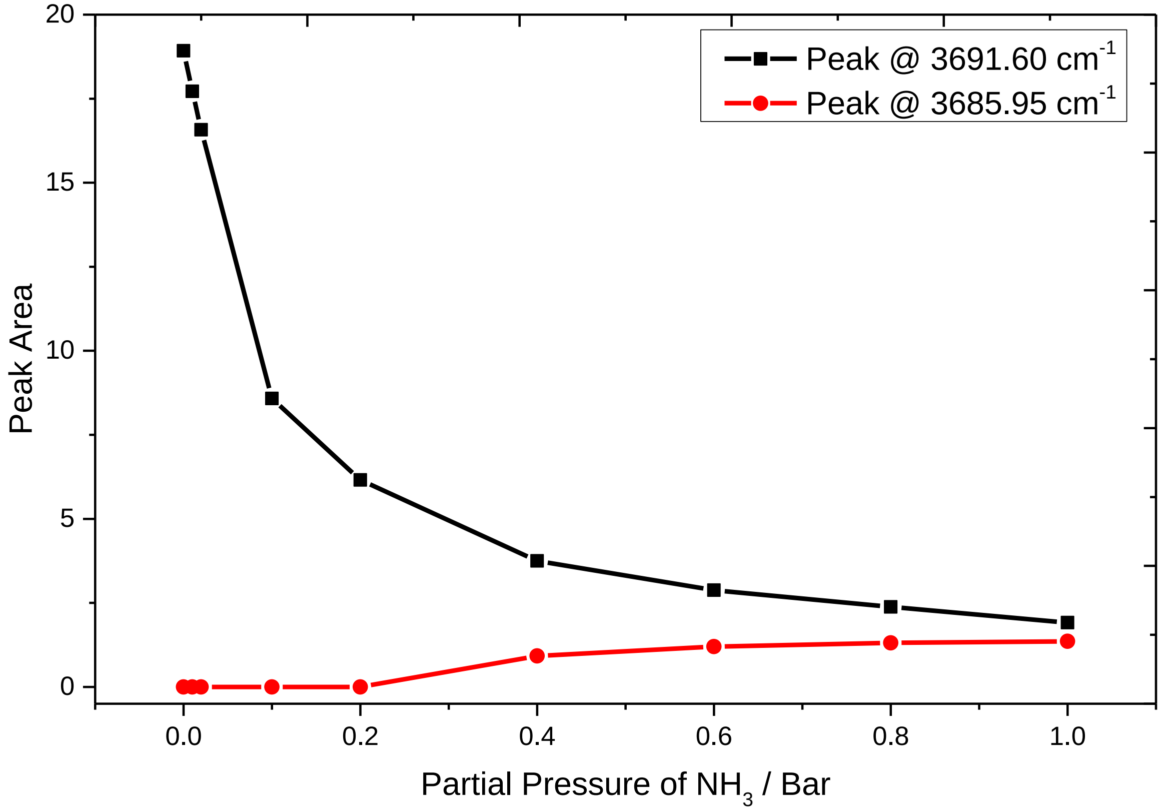


**Figure S15:** Fitted and normalised peak area for the ν(OH) stretching vibration at 3691.60 $cm^{-1}$ and 3685.95 $cm^{-1}$.

## 5. Ammonia Adsorption in Other Porous Materials

**Table S7:** Summary of published materials for ammonia sorption.

| Material | Material Type | Measurement Type | BET surface Area / $m^2 g^{-1}$ | Conditions | Experimental Capacity / $mmol g^{-1}$ | Ammonia Cycling Ability / Regeneration method | Reference |
|---|---|---|---|---|---|---|---|
| CoHCC | Porous Dye | Isotherm | 848 | 298 K, 1 bar | 21.9 | Reversible 150 °C, 24 hrs under vacuum | [5] |
| CuHCF | Porous Dye | Isotherm | 547 | 298 K, 1 bar | 20.2 | Not reversible | [5] |
| $Cu_2Cl_2BBTA$ | MOF | Isotherm | 1205 | 298 K, 1 bar | 19.79 | -* | [6] |
| $Co_2Cl_2BBTA$ | MOF | Isotherm | 1161 | 298 K, 1 bar | 17.95 | 5.6% loss over 3 cycles, 200 °C, under vacuum | [6] |
| Fe-MIL-101-$SO_3H$ | MOF | Isotherm | 1900 | 298 K, 1 bar | 17.8 | -* | [9] |
| BPP-5 | Porous Organic Polymer | Isotherm | 700 | 298 K, 1 bar | 17.7 | -* | [9] |
| BPP-7 | Porous Organic Polymer | Isotherm | 705 | 298 K, 1 bar | 16.1 | -* | [9] |
| $Mn_2Cl_2$(BTDD) | MOF | Isotherm | 1917 | 298 K, 1 bar | 15.47 | Reversible over 3 cycles | [10] |
| COF-10 | COF | Isotherm | 1200 | 298 K, 1 bar | 15 | 4.5% loss over 3 cycles, 200 °C for 12 hr under high vacuum | [7] |
| $Ni_2Cl_2BBTA$ | MOF | Isotherm | 1193 | 298 K, 1 bar | 14.68 | -* | [6] |
| Prussian Blue | Porous Dye | Isotherm | 280 | 298 K, 1 bar | 12.5 | -* | [5] |
| MOF-5 | MOF | Isotherm | 2449 | 298 K, 1 bar | 12.2 | - | [11] |
| MOF-177 | MOF | Isotherm | 3275 | 298 K, 1 bar | 12.2 | - | [11] |
| $Ni_2Cl_2$(BTDD) | MOF | Isotherm | 1762 | 298 K, 1 bar | 12.02 | Reversible over 3 cycles | [10] |
| $Co_2Cl_2$(BTDD) | MOF | Isotherm | 1912 | 298 K, 1 bar | 12 | Reversible over 3 cycles | [10] |
| UiO-66-$NH_3Cl$ | MOF | Isotherm | 730 | 298 K, 1 bar | 12 | Not reversible, $NH_3Cl$ formation blocks pores | [9] |
| MOS-1 | MOF | Isotherm | 1112 | 298 K, 1 bar | 11.5 | Reversible over 5 cycles, vacuum regeneration | [12] |

| Material | Material Type | Measurement Type | BET surface Area / $m^2 g^{-1}$ | Conditions | Experimental Capacity / mmol $g^{-1}$ | Ammonia Cycling Ability / Regeneration method | Reference |
|---|---|---|---|---|---|---|---|
| Amberlyst 15 | Ion-exchange Resin | Isotherm | 225 | 298 K, 1 bar | 11 | -* | [8] |
| UiO-66-$NH_2$ | MOF | Isotherm | 900 | 298 K, 1 bar | 10.5 | -* | [9] |
| MIL-101 | MOF | Isotherm | 3740 | 298 K, 1 bar | 10 | Reversible over 5 cycles, vacuum regeneration | [13] |
| 13X zeolite | Zeolite | Isotherm | 615 | 298 K, 1 bar | 9 | -* | [8] |
| MIL-100 | MOF | Isotherm | 1220 | 298 K, 1 bar | 8 | Reversible over 5 cycles, vacuum regeneration | [13] |
| MCM-41 | Mesoporous Silicate | Isotherm | 990 | 298 K, 1 bar | 7.9 | -* | [7] |
| UiO-66-C | MOF | Isotherm | 800 | 298 K, 1 bar | 7.8 | -* | [14] |
| UiO-66-B | MOF | Isotherm | 780 | 298 K, 1 bar | 6.4 | -* | [14] |
| UiO-66-A | MOF | Isotherm | 820 | 298 K, 1 bar | 5.4 | -* | [14] |
| MIL-53-$NH_2$ | MOF | Isotherm | -* | 298 K, 1 bar | 5.4 | Reversible over 5 cycles, vacuum regeneration | [13] |
| MOS-2 | MOF | Isotherm | 76 | 298 K, 1 bar | 5.2 | Reversible over 5 cycles, vacuum regeneration | [12] |
| MIL-53 | MOF | Isotherm | 945 | 298 K, 1 bar | 4.4 | Reversible over 5 cycles, vacuum regeneration | [13] |
| MOS-3 | MOF | Isotherm | 27 | 298 K, 1 bar | 3.8 | Reversible over 5 cycles, vacuum regeneration | [12] |
| MOF-74-Mg | MOF | Breakthrough | 835 | 298 K, 1 bar | 7.6 | -* | [15] |
| MOF-74-Co | MOF | Breakthrough | 1206 | 298 K, 1 bar | 6.7 | -* | [15] |
| HKUST-1 | MOF | Breakthrough | 1460 | 298 K, 1 bar | 6.6 | 60% reduction over 2 cycles | [16] |
| UiO-66-OH | MOF | Breakthrough | 946 | 298 K, 1 bar | 5.69 | -* | [17] |
| MOF-74-Zn | MOF | Breakthrough | 496 | 298 K, 1 bar | 3.7 | -* | [15] |
| UiO-66-$NH_2$ | MOF | Breakthrough | 1096 | 298 K, 1 bar | 3.56 | -* | [17] |

| **Material** | **Material Type** | **Measurement Type** | **BET surface Area / $m^2$ $g^{-1}$** | **Conditions** | **Experimental Capacity / mmol $g^{-1}$** | **Ammonia Cycling Ability / Regeneration method** | **Reference** |
|---|---|---|---|---|---|---|---|
| MOF-74 -Cu | MOF | Breakthrough | 1170 | 298 K, 1 bar | 3.4 | -* | [18] |
| UiO-66-$(COOH)_2$ | MOF | Breakthrough | 221 | 298 K, 1 bar | 2.83 | -* | [17] |
| MOF-74-Ni | MOF | Breakthrough | 599 | 298 K, 1 bar | 2.3 | -* | [15] |
| UiO-66-$(OH)_2$ | MOF | Breakthrough | 814 | 298 K, 1 bar | 2.29 | -* | [17] |
| UiO-66-$SO_3H$ | MOF | Breakthrough | 323 | 298 K, 1 bar | 2.24 | -* | [17] |
| UiO-66-$NO_2$ | MOF | Breakthrough | 729 | 298 K, 1 bar | 1.98 | -* | [17] |
| UiO-66 | MOF | Breakthrough | 1100–1250 | 298 K, 1 bar | 1.79 | -* | [17] |

*-Information not available

**References**


[1] S. Yang, J. Sun, A. J. Ramirez-Cuesta, S. K. Callear, W. I. David, D. P. Anderson, R. Newby, A. J. Blake, J. E. Parker, C. C. Tang, M. Schroder, *Nat Chem* **2012**, *4*, 887-894.

[2] L. C. Chapon, P. Manuel, P. G. Radaelli, C. Benson, L. Perrott, S. Ansell, N. J. Rhodes, D. Raspino, D. Duxbury, E. Spill, J. Norris, *Neutron News* **2011**, *22*, 22-25.

[3] S. P. Thompson, J. E. Parker, J. Potter, T. P. Hill, A. Birt, T. M. Cobb, F. Yuan, C. C. Tang, *Rev Sci Instrum* **2009**, *80*, 075107.

[4] A. L. Myers, J. M. Prausnitz., *AIChE Journal* **1965**, *11*, 121-127.

[5] A. Takahashi, H. Tanaka, D. Parajuli, T. Nakamura, K. Minami, Y. Sugiyama, Y. Hakuta, S. Ohkoshi, T. Kawamoto, *J Am Chem Soc* **2016**, *138*, 6376-6379.

[6] A. J. Rieth, M. Dincă, *J Am Chem Soc* **2018**, *140*, 3461-3466.

[7] C. J. Doonan, D. J. Tranchemontagne, T. G. Glover, J. R. Hunt, O. M. Yaghi, *Nat Chem* **2010**, *2*, 235-238.

[8] J. Helminen, J. Helenius, E. Paatero, I. Turunen, *J Chem Eng Data* **2001**, *46*, 391-399.

[9] J. F. Van Humbeck, T. M. McDonald, X. Jing, B. M. Wiers, G. Zhu, J. R. Long, *J Am Chem Soc* **2014**, *136*, 2432-2440.

[10] A. J. Rieth, Y. Tulchinsky, M. Dinca, *J Am Chem Soc* **2016**, *138*, 9401-9404.

[11] D. Saha, S. Deng, *J Colloid Interface Sci* **2010**, *348*, 615-620.

[12] Y. Chen, Y. Wang, C. Yang, S. Wang, J. Yang, J. Li, *ACS Sustain Chem Eng* **2017**, *5*, 5082-5089.

[13] Y. Chen, F. Zhang, Y. Wang, C. Yang, J. Yang, J. Li, *Microporous Mesoporous Mater* **2018**, *258*, 170-177.

[14] W. Morris, C. J. Doonan, O. M. Yaghi, *Inorg Chem* **2011**, *50*, 6853-6855.

[15] T. Grant Glover, G. W. Peterson, B. J. Schindler, D. Britt, O. Yaghi, *Chem Eng Sci* **2011**, *66*, 163-170.

[16] G. W. Peterson, G. W. Wagner, A. Balboa, J. Mahle, T. Sewell, C. J. Karwacki, *J Phys Chem C* **2009**, *113*, 13906-13917.

[17] H. Jasuja, G. W. Peterson, J. B. Decoste, M. A. Browe, K. S. Walton, *Chem Eng Sci* **2015**, *124*, 118-124.

[18] M. J. Katz, A. J. Howarth, P. Z. Moghadam, J. B. DeCoste, R. Q. Snurr, J. T. Hupp, O. K. Farha, *Dalton Trans* **2016**, *45*, 4150-4153.
